# Socioeconomic Disparities in Allogeneic Transplant Access Within a Publicly Funded Healthcare System

**DOI:** 10.1002/jha2.70172

**Published:** 2025-10-27

**Authors:** Stephen P. Hibbs, Yosef Joseph Rene Amel Riazat‐Kesh, Funmi Oyesanya, Matthew L. Smith, Jeff K. Davies

**Affiliations:** ^1^ Wolfson Institute of Population Health Queen Mary University of London London UK; ^2^ Barts Health NHS Trust London UK; ^3^ New York Presbyterian Hospital New York City New York USA; ^4^ Centre for Haemato‐Oncology, Barts Cancer Institute Queen Mary University of London London UK

1

Sociodemographic disparities in access to and outcomes from allogeneic haematopoietic stem cell transplants (HSCT) are well‐documented, particularly in the USA [1]. In the USA context, the absence of universal access to specialist healthcare and the close association between racial minoritisation and socioeconomic deprivation create significant barriers to equitable care. With a different history of racial and ethnic minoritisation and a National Health Service (NHS) that offers free healthcare at the point of use, the UK provides a contrasting context to examine access to allogeneic HSCT.

Despite this different context, a recent UK study across 13,978 transplants found that patient ethnicity predicted survival following allogeneic HSCT receipt [2]. The authors noted that their analysis was limited to patients who received HSCT, preventing assessment of potential inequities in access. To our knowledge, no UK studies have examined the impact of sociodemographic factors on access to allogeneic HSCT.

We present our data assessing how sociodemographic factors influence the likelihood of receiving HSCT for patients with acute leukaemia in the UK. Beyond donor availability and universal health coverage, we suggest other factors as to why patients do or do not receive HSCT, including the decisions of doctors and patients themselves.

In 2021, we conducted a retrospective review at our transplant centre (St Bartholomew's Hospital, London, UK), examining 260 cases of acute leukaemia identified from MDT meeting minutes. These meetings included all new cases diagnosed across the regional network and transplants completed over the preceding three years. For this analysis, we included all patients discussed between 2018 and 2019, of whom 112 (43%) received allogeneic HSCT. We excluded six patients with lymphoblastic lymphoma (LBL) without bone marrow involvement, in whom HSCT would rarely be indicated, leaving 254 in the analytical cohort. Demographics for patients included in the analysis are shown in Table 1. All statistical analyses were conducted using Python 3.8.

**TABLE 1:** Patient demographics.

		**All patients**	**Transplanted**		**Not transplanted**	
**Characteristic**			Number	%	Number	%
Total number		254	112	44%	142	56%
Diagnosis						
	AML	170	70	41%	100	59%
	AML/MDS	23	6	26%	17	74%
	ALL	56	32	57%	24	43%
	MPAL	4	3	75%	1	25%
	LBL	1	1	100%	0	0%
Gender						
	Female	109	48	44%	61	56%
	Male	145	64	44%	81	56%
Ethnicity						
	White British	114	47	41%	67	59%
	White Other	24	12	50%	12	50%
	Asian	32	16	50%	16	50%
	Black	21	9	43%	12	57%
	Mixed	3	3	100%	0	0%
	Other	52	21	40%	31	60%
HCT‐CI Risk Group						
	Low	145	75	52%	70	48%
	Intermediate	56	22	39%	34	61%
	High	49	15	31%	34	69%
Baseline disease risk						
	Poor	99	49	49%	50	51%
	Intermediate	108	53	49%	55	51%
	Good	44	9	20%	35	80%
IMD decile ranking						
	1st	10	4	40%	6	60%
	2nd	29	11	38%	18	62%
	3rd	48	12	25%	36	75%
	4th	35	13	37%	22	63%
	5th	25	15	60%	10	40%
	6th	24	11	46%	13	54%
	7th	25	12	48%	13	52%
	8th	23	13	57%	10	43%
	9th	19	13	68%	6	32%
	10th	16	8	50%	8	50%

Using multivariable logistic regression, we analysed the odds of receiving HSCT, adjusting for age, HCT‐comorbidity index (HCT‐CI), and baseline disease risk [3, 4] (Table 2). Our findings showed that living in a wealthier area (measured by the index of multiple deprivation, IMD) was associated with higher odds of receiving HSCT (odds ratio 1.24, 95% CI 1.11–1.38). Conversely, patients living in more deprived areas (by IMD decile) had lower odds of receiving HSCT. Ethnicity was not associated with odds of HSCT receipt within this limited model.

**TABLE 2:** Multivariate analysis of factors associated with receipt of HSCT.

	**Odds ratio (95% Confidence Intervals)**
Age	0.94 (0.92–0.96)
Index of Multiple Deprivation (IMD) rank	1.24 (1.11–1.38)
Disease risk	1.97 (1.3–2.97)
Comorbidity risk (HCT‐CI)	1.12 (0.77–1.64)

*Note*: The dependent variable is whether an individual received HSCT. Index of Multiple Deprivation (IMD) rank assigned according to the English Indices of Deprivation 2019 data on opendatacommunities.org – a higher IMD rank signifies a less deprived area. Disease Risk: assigned based on Döhner et al. [3] for patients with acute myeloid leukaemia and Terwilliger et al. [4] for patients with acute lymphoblastic leukaemia. Proportionality of odds and interactions between variables were not assessed. HCT‐CI: haematopoietic cell transplantation (HCT)‐specific comorbidity index.

Given that all patients were treated within a publicly funded healthcare system, it is notable that individuals from wealthier areas were more likely to receive HSCT than those from more deprived areas. Our methods could not determine whether the differences in HSCT receipt among individuals of varying socioeconomic status were due to donor availability, referral patterns, fitness assessments, or patient decision‐making. The latter two factors warrant further reflection and consideration.

Transplant doctors assess whether a patient is fit enough to undergo cellular therapies. These evaluations often involve subjective judgments of who is a ‘transplant candidate’ which may be shaped by socio‐demographic factors such as ethnicity or socio‐economic status. For example, a patient arriving to a clinical assessment in a wheelchair may be perceived as unfit by their doctor—particularly if they are meeting for the first time. However, for some families, the decision to use a wheelchair in hospital may have a different meaning to that of the doctor, signifying personal suffering and family support rather than severe functional impairment. Such misinterpretations could disadvantage patients from certain groups during fitness decision‐making.

When a patient chooses whether to proceed with cellular therapies, their decision‐making process is influenced by many factors. These include trust in healthcare systems, availability of support networks, financial and family responsibilities, and whether they are enabled to communicate effectively during consultations [5].

There are several limitations to our study. The IMD is a robust, government‐endorsed measure that integrates multiple data sources to evaluate deprivation at a small‐area level across the UK. However, it reflects aggregated characteristics of geographic areas rather than individuals and thus may not accurately represent the socioeconomic status of every patient. In addition, socioeconomic status intersects with other factors—such as health literacy, housing insecurity, and English proficiency—that may influence the likelihood of receiving HSCT. These variables were inconsistently documented in clinical records, preventing their inclusion in our analysis, but warrant further investigation.

Clinician and patient decision‐making factors intersect with socio‐demographic groupings and warrant closer examination. Further investigation across cellular therapy assessments, decisions, referrals and receipt is necessary and may illuminate factors that are directly amenable to change.

## Author Contributions

SPH, YJRAR‐K, FO and MLS conceptualised the project. YJRAR‐K collected and analysed data. JKD provided supervision. SPH wrote the initial draft, and YJRAR‐K, FO, MLS and JKD critically reviewed and edited the manuscript.

## Funding

Stephen P. Hibbs is supported by a HARP doctoral research fellowship, funded by the Wellcome Trust (Grant number 223500/Z/21/Z). No funding was received for this study.

## Ethics Statement

This study was conducted as a service evaluation project, designed to assess and improve existing clinical practice. As such, it did not require review by a research ethics committee.

## Consent

The authors have nothing to report.

## Conflicts of Interest

The authors declare no conflicts of interest.

## Data Availability

The data that support the findings of this study are not publicly available due to inclusion of sensitive demographic information.
